# RhoE/ROCK2 regulates chemoresistance through NF-κB/IL-6/ STAT3 signaling in hepatocellular carcinoma

**DOI:** 10.18632/oncotarget.9441

**Published:** 2016-05-18

**Authors:** Wei Ma, Karen Man-Fong Sze, Lo Kong Chan, Joyce Man-Fong Lee, Larry Lai Wei, Chun-Ming Wong, Terence Kin-Wah Lee, Carmen Chak-Lui Wong, Irene Oi-Lin Ng

**Affiliations:** ^1^ Department of Pathology and State Key Laboratory for Liver Research, The University of Hong Kong, Pokfulam, Hong Kong

**Keywords:** Rho GTPase, Rho-associated kinase, HCC, drug-resistance, cell survival

## Abstract

Small Rho GTPase (Rho) and its immediate effector Rho kinase (ROCK) are reported to regulate cell survival, but the detailed molecular mechanism remains largely unknown. We had previously shown that Rho/ROCK signaling was highly activated in hepatocellular carcinoma (HCC). In this study, we further demonstrated that downregulation of RhoE, a RhoA antagonist, and upregulation of ROCK enhanced resistance to chemotherapy in HCC in both *in vitro* cell and *in vivo* murine xenograft models, whereas a ROCK inhibitor was able to profoundly sensitize HCC tumors to cisplatin treatment. Specifically, the ROCK2 isoform but not ROCK1 maintained the chemoresistance in HCC cells. Mechanistically, we demonstrated that activation of ROCK2 enhanced the phosphorylation of JAK2 and STAT3 through increased expression of IL-6 and the IL-6 receptor complex. We also identified IKKβ as the direct downstream target of Rho/ROCK, and activation of ROCK2 significantly augmented NF-κB transcription activity and induced IL-6 expression. These data indicate that Rho/ROCK signaling activates a positive feedback loop of IKKβ/NF-κB/IL-6/STAT3 which confers chemoresistance to HCC cells and is a potential molecular target for HCC therapy.

## INTRODUCTION

Hepatocellular carcinoma (HCC) is the seventh most common cancer worldwide and ranks the third leading cause of cancer-related mortality [1]. HCC is generally resistant to systemic chemotherapy which highly limits treatment options to patients. On the other hand, sorafenib is the only molecularly targeted drug approved for treating patients with advanced HCC. However, sorafenib only extends the median overall survival of patients by about 3 months [2]. Cancer stem cells (CSCs), activation of pro-survival pathways and hypoxia are important molecular mechanisms contributing to the highly chemoresistant nature of HCC. For example, Ma et al. has demonstrated that CD133^+^ HCC CSCs can activate Akt in order to enhance their resistance to chemotherapy treatment [3]. In addition, the large hypoxic core of HCC tumors also confers higher survival ability of HCC cells. Hypoxia stabilizes transcription factor HIF-1α and HIF-1α promotes expression of pro-survival gens such as surviving and Bcl-2 [4] and drug-resistant genes such as MDR1 and P-glycoprotein [5]. Therefore there is an urgent need to identify new therapeutic targets to combat against chemoresistance in HCC and provide patients with more treatment choices.

The family of small Rho GTPases is a subfamily of the Ras superfamily. While members of the Rho GTPases are most well-known for their functions in regulating cytoskeleton remodeling events, they are also reported to be involved in controlling apoptotic signaling and cell survival [6]. RhoA and its effector Rho kinase (ROCK) are reported to enhance cell survival and confer resistance to chemotherapy in different cancer types [7, 8]. However, the exact roles of Rho/ROCK in regulating HCC chemoresistance remain undiscovered and the detailed underlying mechanism remains largely unknown.

Both JAK/STAT3 and NF-κB pathways have been extensively studied for their functions in promoting cell survival through activating transcription of many pro-survival genes [9, 10]. Rho/ROCK has been shown to interact with JAK/STAT3. Activation of RhoA can upregulate the phosphorylation of STAT3 at Y705 and S727 [11] and induce activation of NF-κB [12]. Therefore there is a possibility that Rho/ROCK regulates chemoresistance via the JAK/STAT3 and NF-κB pathways in HCC.

We had previously demonstrated that ROCK2 was overexpressed and RhoE, an antagonist of the Rho/ROCK signaling, was underexpressed in HCC and together they promote HCC metastasis [13, 14]. In this study, we identified that these two events resulted in hyper-activation of ROCK2, which in turn activated an IKKβ/NF-κB/IL-6/STAT3 positive feedback loop and conferred chemoresistance to HCC cells. Our work also led to the finding of ROCK inhibitor as a potential therapeutic agent for both chemo-sensitization and suppression of metastasis in HCC.

## RESULTS

### RhoE/ROCK2 modulates chemoresistance in HCC cells

**Figure 1:**
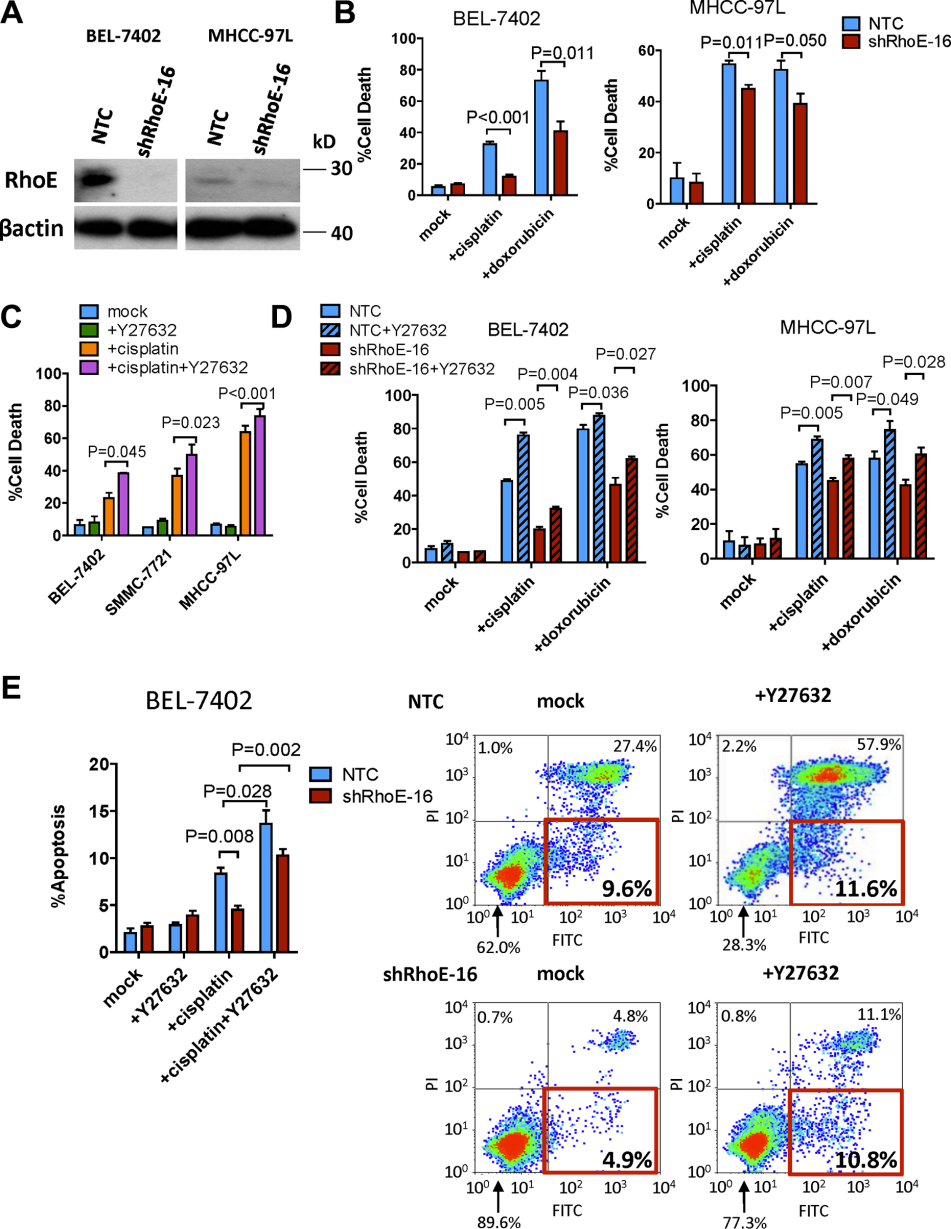
ROCK activity regulated chemoresistance in HCC cells (**A**) Western blot showing RhoE protein levels in both non-target control and shRhoE-16 infected HCC cells. (**B**, **C** and **D**) Quantification of percentage of cell death induced by (B) 48-hour treatment of cisplatin and doxorubicin in non-target control (NTC) and RhoE knockdown cells, (C) 48-hour treatment of i) mock, ii) cisplatin, iii) Y27632, and iv) cisplatin and Y27632. (D) 48-hour treatment of cisplatin and doxorubicin with or without Y27632 in NTC and RhoE knockdown cells. (**E**) Annexin V flow cytometry assessment of the apoptotic cell population after 24 hours of cisplatin treatment with or without Y27632. Cells undergoing apoptosis are represented by the Annexin V^+ve^/PI^−ve^ populations (red rectangles). In all panels, 3 experiments are represented. *P*-value was calculated using Student's *t* test. 20 ug/ml cisplatin and 10 μM Y27632 were used.

To investigate the effect of Rho/ROCK signaling on chemoresistance in HCC cells, we first knocked down its antagonist RhoE by short-hairpin RNA (shRNA) approach in BEL-7402 and MHCC-97L HCC cells (Figure 1A). The cells were then treated with two different chemotherapeutic drugs, cisplatin and doxorubicin, that are used in HCC patients, respectively. Knockdown of RhoE repressed the chemodrug-induced cell death as compared with the non-target control (NTC) (Figure 1B). Next, we examined if this effect was related to ROCK activity. Addition of ROCK inhibitor, Y27632, to BEL-7402, MHCC-97L and SMMC-7721 cells significantly sensitized them to cisplatin treatment (Figure 1C). Moreover, the cell-death suppression effect resulted from RhoE knockdown (as seen from Figure 1A) was also rescued by co-treatment with Y27632 for 48 hours (Figure 1D). An immortalized normal liver cell line LO2 was tested for the toxicity of combined treatment of cisplatin and Y27632. As expected, knockdown of RhoE by siRNA enhanced resistance to cisplatin in LO2 cells (Supplementary Figure 1A). Although co-treatment of cisplatin and Y27632 also had increased toxicity in LO2 cells, the degree of sensitization is less as compared with that in BEL-7402 and MHCC-97L cells (Supplementary Figure 1B). This suggests that Y27632 sensitizes HCC cells to cisplatin treatment more than normal hepatocytes. Since Rho/ROCK signaling was reported to regulate cell survival via modulating the expression of anti-apoptotic genes [7, 15], we investigated if cisplatin-induced apoptosis could be altered by Rho/ROCK in BEL-7402. Annexin-V staining flow cytometry was performed to measure the apoptotic cell population induced by cisplatin treatment in RhoE-knockdown cells with or without the addition of Y27632 for 24 hours. The results demonstrated that downregulation of RhoE dramatically reduced the apoptotic population of cells (Annexin-V^+ve^, PI^−ve^) treated with cisplatin alone as compared with that of NTC. Reciprocally, co-treating the cells with Y27632 restored cisplatin-induced apoptosis (Figure 1E).

**Figure 2:**
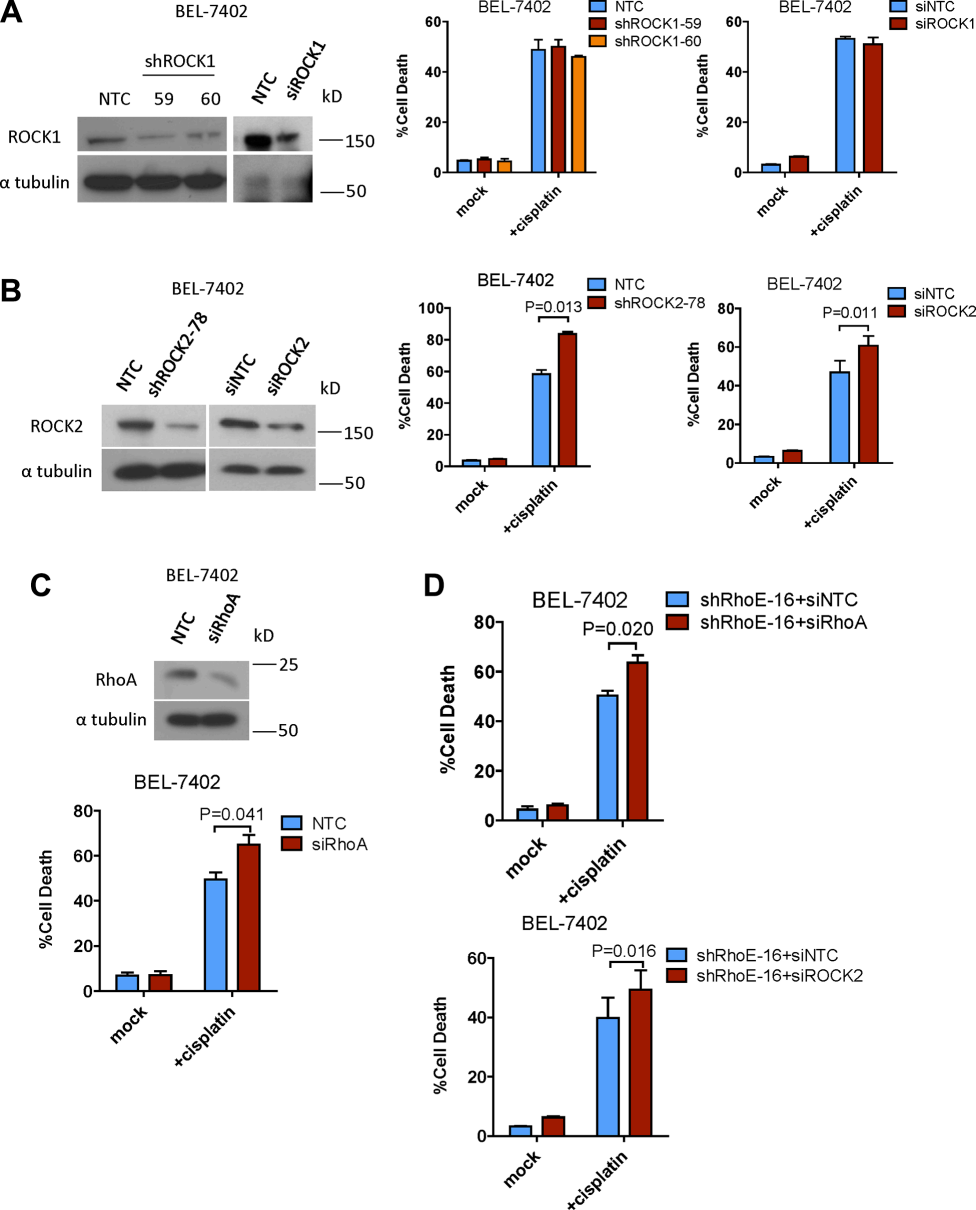
Inhibition of ROCK2 but not ROCK1 sensitized HCC cells to chemotherapy (**A** and **B**) Western blot analysis was used to confirm the successful knockdown of ROCK1/2 by both sh- and si-RNA. The amount of cell death 48 hours after treatment with cisplatin was quantified. Only knockdown of ROCK2 enhanced cisplatin-induced cell death. (**C**) Knockdown of the RhoA protein by siRNA approach was confirmed by Western blot analysis. The amount of cell death 48 hours after treatment with cisplatin was quantified. RhoA knockdown sensitized HCC cells to cisplatin treatment. (**D**) Knockdown of RhoA and ROCK2 rescued the enhanced chemoresistance induced by RhoE knockdown in HCC cells. In all panels, 3 experiments are represented. *P*-value was calculated using Student's *t* test.

ROCK has two isoforms, ROCK1 and ROCK2. Previous studies have suggested that these two isoforms have differential functions [16]. Therefore we aimed to find out which isoform of ROCK was responsible for contributing to cell survival in HCC cells. We knocked down ROCK1 and ROCK2 in BEL-7402 with siRNA and shRNA, respectively, and then treated the cells with cisplatin. Knockdown of ROCK1 had no observable effects on cisplatin-induced cell death (Figure 2A). In contrast, knockdown of ROCK2 significantly sensitized BEL-7402 to cisplatin (Figure 2B). This suggests that ROCK2 is able to regulate cell survival in HCC cells. Furthermore, since RhoE is reported to suppress ROCK2 activity by repressing RhoA, we knocked down RhoA by siRNA and found that RhoA knockdown also sensitized BEL-7402 cells to cisplatin (Figure 2C). Of note, knockdown of RhoA and ROCK2, respectively, was able to rescue the chemoresistance enhanced by RhoE knockdown (Figure 2D).

To ensure that the effect we observed with Y27632 was not due to non-specific activity of the inhibitor, we overexpressed ROCK2 in SMMC-7721 and BEL-7402 cells using the transcription activator-like effectors (TALE)-linked transcription factor approach, as described [17] with slight modification (Supplementary Figure 2A and 2B). As expected, cells with ROCK2 overexpression showed enhanced chemoresistance and reduced apoptosis to cisplatin treatment as compared with the vector control (Supplementary Figure 2C and 2D). Taken together, these data strongly suggest that RhoE/ROCK2 modulates chemoresistance in HCC *in vitro*.

### RhoE/ROCK2 regulates chemoresistance of HCC *in vivo*

**Figure 3:**
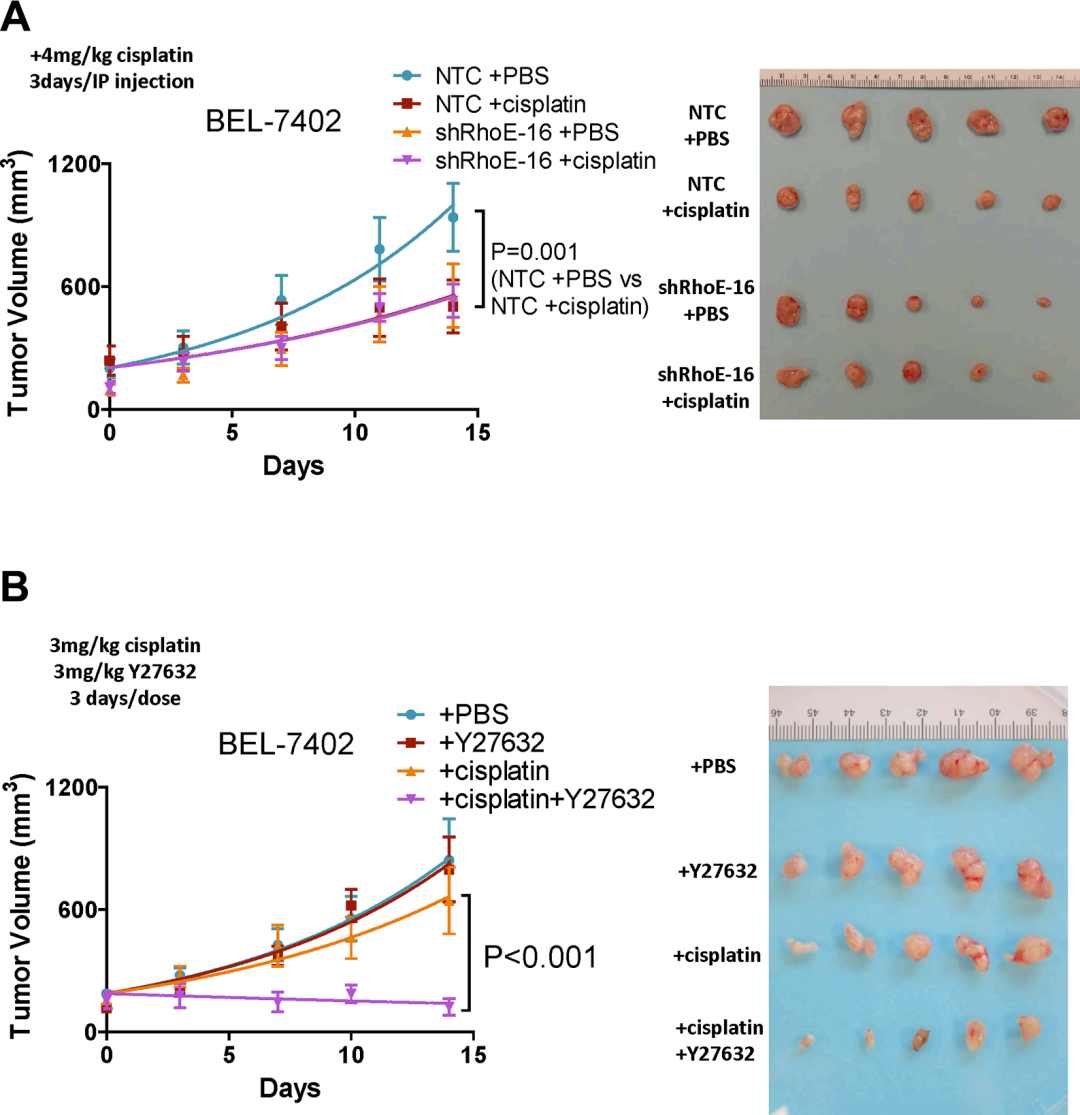
Rho/ROCK activity modulates HCC chemoresistance *in vivo* (**A** and **B**) Xenograft tumor growth derived from subcutaneously injected HCC cells in nude mice. Tumors were first allowed to grow to about 100 mm^3^ in size and then different treatments were given to the mice as described below. (A) RhoE knockdown BEL-7402 cells and NTC were used. Mice were given either 4 mg/kg cisplatin or PBS through intraperitoneal injection once every 3 days. Cisplatin only slowed down the growth of NTC tumors but not that of RhoE knockdown tumors. (B) Parental BEL-7402 cells were used. Mice were given i) PBS, ii) 4 mg/kg Y27632, iii) 3 mg/kg cisplatin, and iv) 3 mg/kg cisplatin + 4 mg/kg Y27632, respectively, through intraperitoneal injection once every 3 days. Combined treatment of cisplatin and Y27632 had synergistic effect in suppressing HCC tumor growth than cisplatin alone. Representative result from 3 experiments is shown. *P*-value was calculated using non-linear regression.

To investigate if the effects of RhoE/ROCK2 on cell survival we observed *in vitro* also occurred *in vivo*, we first used a subcutaneous injection model in immune-deficient nude mice. RhoE knockdown BEL-7402 cells or NTC were injected subcutaneously into nude mice and the tumors were allowed to grow to about 100 mm^3^ in size. We then administrated the mice with either cisplatin or phosphate buffered saline (PBS) and the tumor growth rate was recorded. Tumors derived from NTC cells responded well to cisplatin treatment whereas tumors derived from RhoE knockdown cells displayed similar growth rates between the PBS and cisplatin treatment groups (Figure 3A). The same experiment was performed using ROCK2-overexpressing BEL7402 cells. In both ROCK2-overexpressing clones, there was no significant difference between the tumor growth rates of the cisplatin- and PBS-treated mice. On the other hand, cisplatin treatment drastically inhibited tumor growth in vector control cells (Supplementary Figure 3A). We also tested the chemoresistance of the NTC and RhoE knockdown HCC cells in an orthotopic liver injection model. MHCC-97L cells were used in this experiment since SMMC-7721 does not grow at all and BEL-7402 does not grow well in in the orthotopic liver injection model. Consistent with the results in the subcutaneous model, cisplatin profoundly diminished the tumor volume in the NTC group, while it exerted no observable effects in the RhoE knockdown group (Supplementary Figure 3B). It should be noted that both knockdown of RhoE and overexpression of ROCK2 suppressed HCC growth. This was in line with the results from other groups [18], suggesting activation of Rho/ROCK signaling has growth suppressive effect in HCC. This effect may be due to the interaction between the adhesive contexts in the local microenvironment and the cytoskeletal tension brought by Rho/ROCK activation [19].

In the hope that ROCK inhibitor may serve as a putative chemo-sensitizing therapy in treating HCC, we examined the effect of Y27632 in combination with cisplatin. Subcutaneous injection model was performed as mentioned above using parental BEL-7402 cells. The mice were divided into four groups and received i) PBS, ii) Y27632, iii) cisplatin, and iv) cisplatin+Y27632, respectively. A lower concentration of cisplatin (3 mg/kg) was used in this experiment comparing to the dose (4 mg/kg) used in Figure 3A in order to clearly show the effect of Y27632 on sensitizing HCC cells to cisplatin. Treatment of Y27632 alone showed no difference in tumor growth rate compared to PBS. Cisplatin slightly suppressed the tumor growth while the combined treatment of cisplatin and Y27632 had remarkable synergistic effect of suppressing tumor growth (Figure 3B). In addition, we also tested the effect of the combined treatment of cisplatin and Y27632 in an orthotopic model using parental MHCC-97L cells. Only cisplatin+Y27632 could significantly inhibit tumor growth in the orthotopic model, although the effect was not as drastic as in the subcutaneous model (Supplementary Figure 3C). It should be noted that in previous report, Y27632 was given into the peritoneal cavity with micro-osmotic pumps, which then ensured a more stable dose and hence persistent effect [20]. Nonetheless, collectively, these findings indicate that ROCK inhibitor exhibits chemo-sensitizing effects *in vivo* in HCC.

### Enhanced ROCK2 activity promotes the activation of JAK2/STAT3

**Figure 4:**
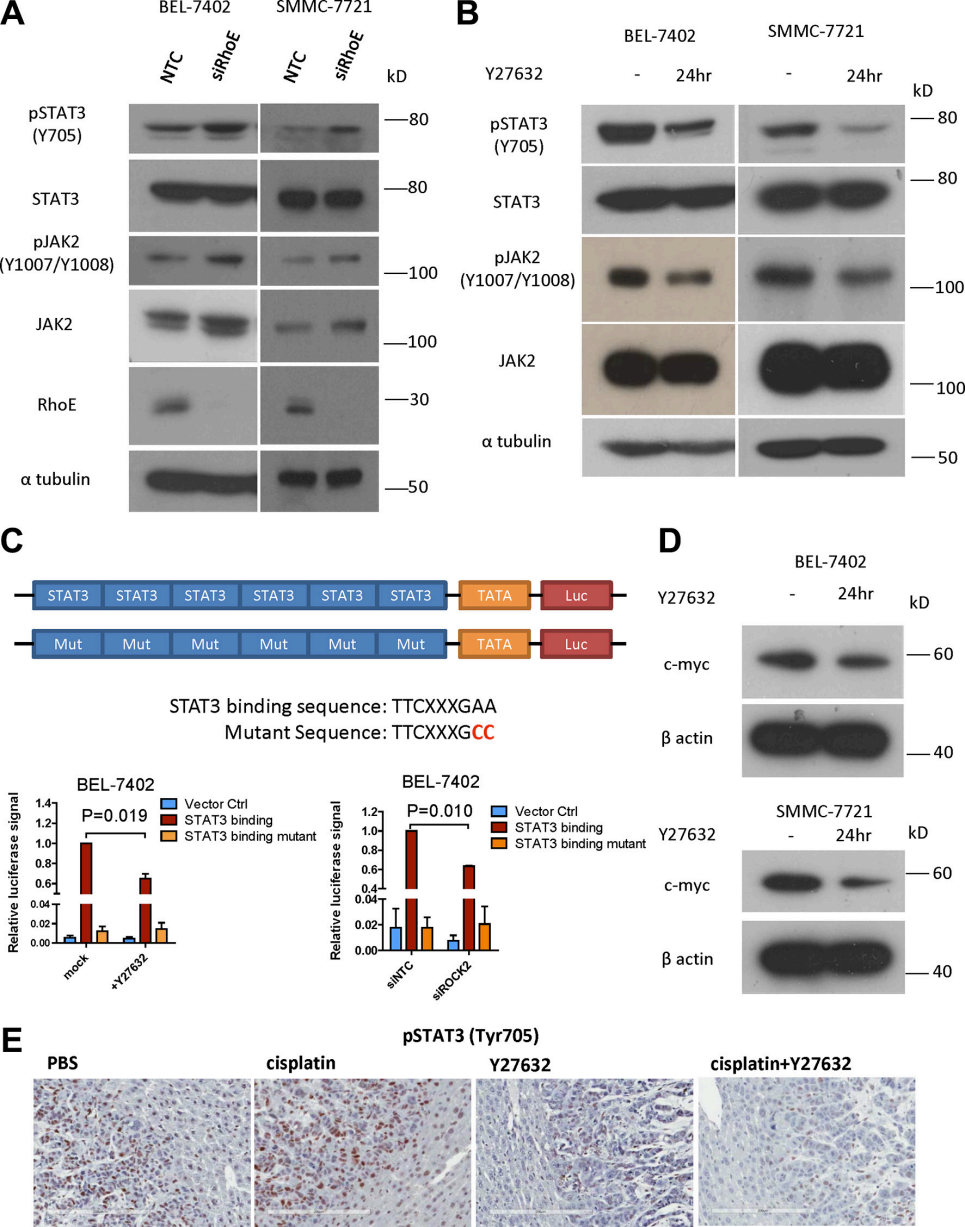
Inhibition of ROCK repressed the activity of JAK2 and STAT3 (**A**) Knockdown of RhoE by siRNA induced phosphorylation of both JAK2 and STAT3 in BEL-7402 and SMMC-7721 cells. (**B**) Treatment with Y27632 for 24 hours inhibited the phosphorylation of JAK2 and STAT3 in BEL-7402 and SMMC-7721 cells. (**C**) Schematic diagram showing the construct which expressed firefly luciferase under the control of STAT3-binding elements. Both Y27632 and ROCK2 knockdown by siRNA reduced the luciferase activity in BEL-7402 cells. SV40-driven expression of Renilla luciferase was used as normalization control. (**D**) Treatment with Y27632 for 24 hours decreased the protein level of c-myc, a transcriptional target of STAT3, in BEL-7402 and SMMC-7721 cells. (**E**) Representative IHC staining of p-STAT3 (Y705) in xenograft tumors harvested from subcutaneous tumor injection model (Figure 3B). Y27632 remarkably diminished the p-STAT3 (Y705) signals; the p-STAT3 stain was nearly totally abolished in the combined Y27632 and cisplatin treatment group. Original magnification, ×20; scale bars: 200 μm. 10 μM Y27632 was used. In all panels, 3 experiments are represented. *P*-value was calculated using Student's *t* test.

In order to elucidate the underlying molecular mechanism of how RhoE/ROCK2 might regulate HCC chemoresistance, we screened a number of pro-survival signaling pathways and identified JAK2/STAT3 as a candidate. With Western blotting, we found that RhoE knockdown by siRNA in BEL-7402 and SMMC-7721 enhanced the phosphorylation of both JAK2 and STAT3 (Figure 4A). Consistently, addition of Y27632 repressed the phosphorylation of JAK2 and STAT3 (Figure 4B). Furthermore, phosphorylation of STAT3 was also upregulated in ROCK2-overexpressing BEL-7402 cells when compared to the vector control (Supplementary Figure 4).

STAT3 is a transcription factor which regulates the transcription of pro-survival genes such as survivin, c-myc and cyclin D [9]. To validate that the transcription activity of STAT3 was upregulated by ROCK2, we constructed a vector expressing firefly luciferase under the control of 6× STAT3 binding elements. Using this construct, we showed that both ROCK inhibitor and knockdown of ROCK2 by siRNA decreased the STAT3-mediated transcription of firefly luciferase (Figure 4C). Using Western blotting, we also observed that one of the target genes of STAT3, c-myc, was downregulated after addition of Y27632 to HCC cells (Figure 4D). Furthermore, we stained for p-STAT3 in the tumors harvested from the subcutaneous injection model in Figure 3B. We found strong nuclear stain of p-STAT3 in both the PBS and cisplatin-alone groups. In contrast, the p-STAT3 level was markedly abrogated in both Y27632-alone and cisplatin+Y27632 groups. In particular, the p-STAT3 stain was nearly totally abolished in cisplatin+Y27632 group (Figure 4E). The data suggest that RhoE/ROCK2 modulates chemoresistance in HCC via the JAK2/STAT3 pathway. Indeed, addition of JAK2 inhibitor AG490 to BEL-7402 cells repressed phosphorylation of STAT3 and sensitized the cells to cisplatin treatment in a dose-dependent manner (Supplementary Figure 5A) and addition of AG490 was able to rescue the enhanced chemoresistance in ROCK2 overexpressing BEL-7402 cells (Supplementary Figure 5B).

### RhoE/ROCK2 regulates the expression of IL-6 and IL-6 receptor

**Figure 5:**
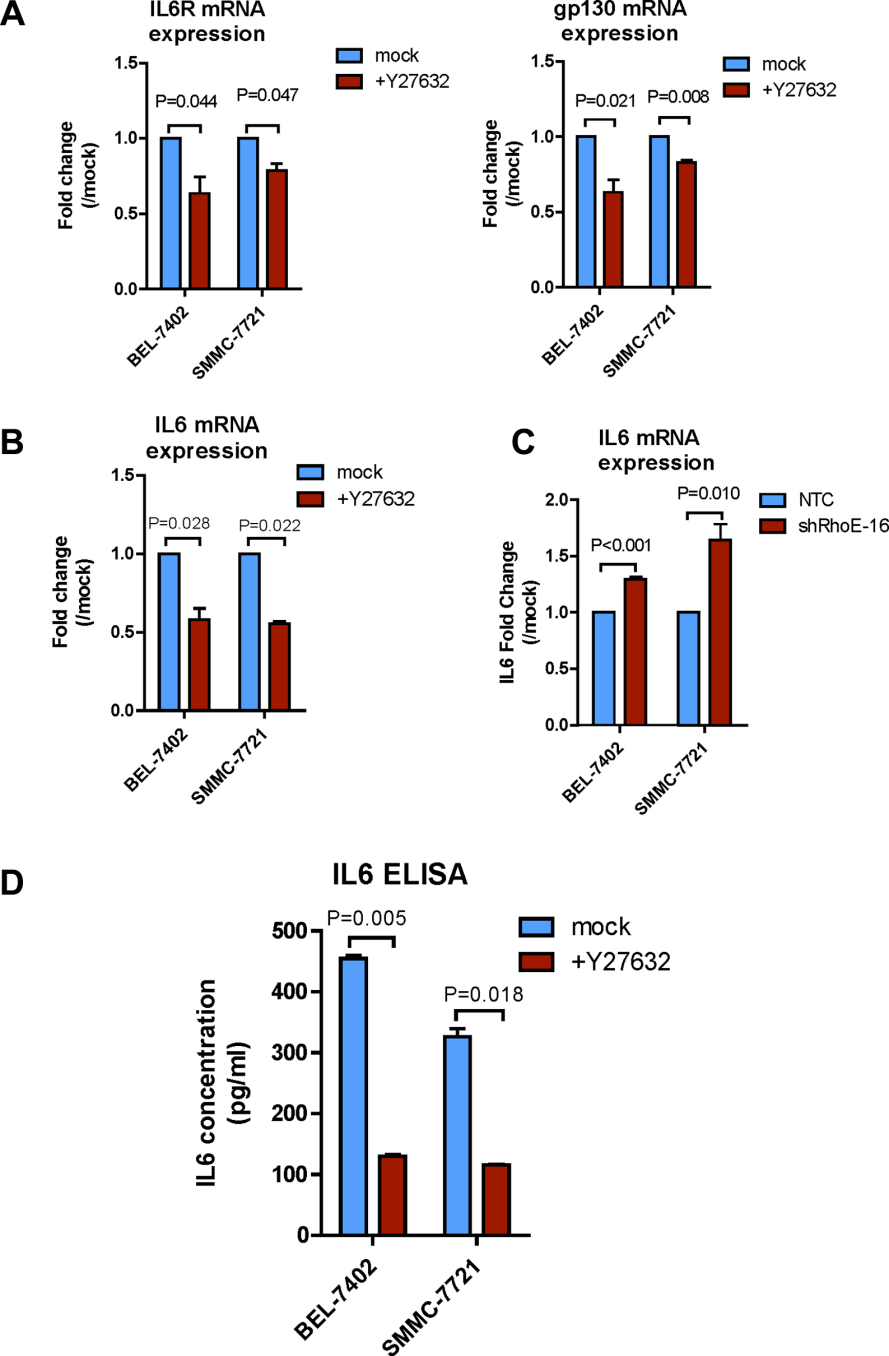
Rho/ROCK signaling regulates expressions of IL-6 receptor and IL-6 (**A** and **B**) Real-time qPCR was used to measure the mRNA levels of (A) IL-6R and gp130, and (B) IL-6 in mock and Y7632-treated BEL-7402 and SMMC-7721 cells. Y27632-treated cells had reduced mRNA expression of IL-6R, gp130 and IL-6. (**C**) Knockdown of RhoE significantly augmented the mRNA expression of IL-6 in BEL-7402 and SMMC-7721 cells as compared to the corresponding NTCs. (**D**) ELISA assay was used to assess IL-6 secretion to the cultured medium by BEL-7402 and SMMC-7721 cells. Inhibition of ROCK by 24-hour treatment of Y27632 markedly repressed IL-6 secretion. 10 μM Y27632 was used. In all panels, 3 experiments are represented. *P*-value was calculated using Student's *t* test.

To explore the mechanism how JAK2/STAT3 were activated by ROCK2, we used Y27632 and then examined the expression of the IL-6 receptor complex and IL-6. After treating with Y27632 for 24 hours, the mRNA expression levels of IL-6R, gp130 and IL-6 were all significantly reduced in SMMC-7721 and BEL-7402 cells (Figure 5A and 5B). Reciprocally, knockdown of RhoE enhanced the IL-6 mRNA expression when compared with the corresponding NTCs (Figure 5C). To further confirm that the alteration in ROCK signaling indeed affected the secretion of functional IL-6, we used IL-6-specific ELISA assay to detect IL-6 level in the HCC-cell-conditioned media. We found that after incubating SMMC-7721 and BEL-7402 cells with Y27632 for 24 hours, level of IL-6 in each of the conditioned media was dramatically reduced as compared to the mock controls (Figure 5D). Consistently, the IL-6 level in the conditioned medium of the ROCK2-overexpressing cells showed significant increases as compared with that of the vector control cells (Supplementary Figure 6). Altogether these data strongly indicate that RhoE/ROCK2 regulate the expression levels of IL-6 and IL-6 receptor.

### Rho/ROCK signaling targets IL-6/JAK2/STAT3 through NF-κB

**Figure 6:**
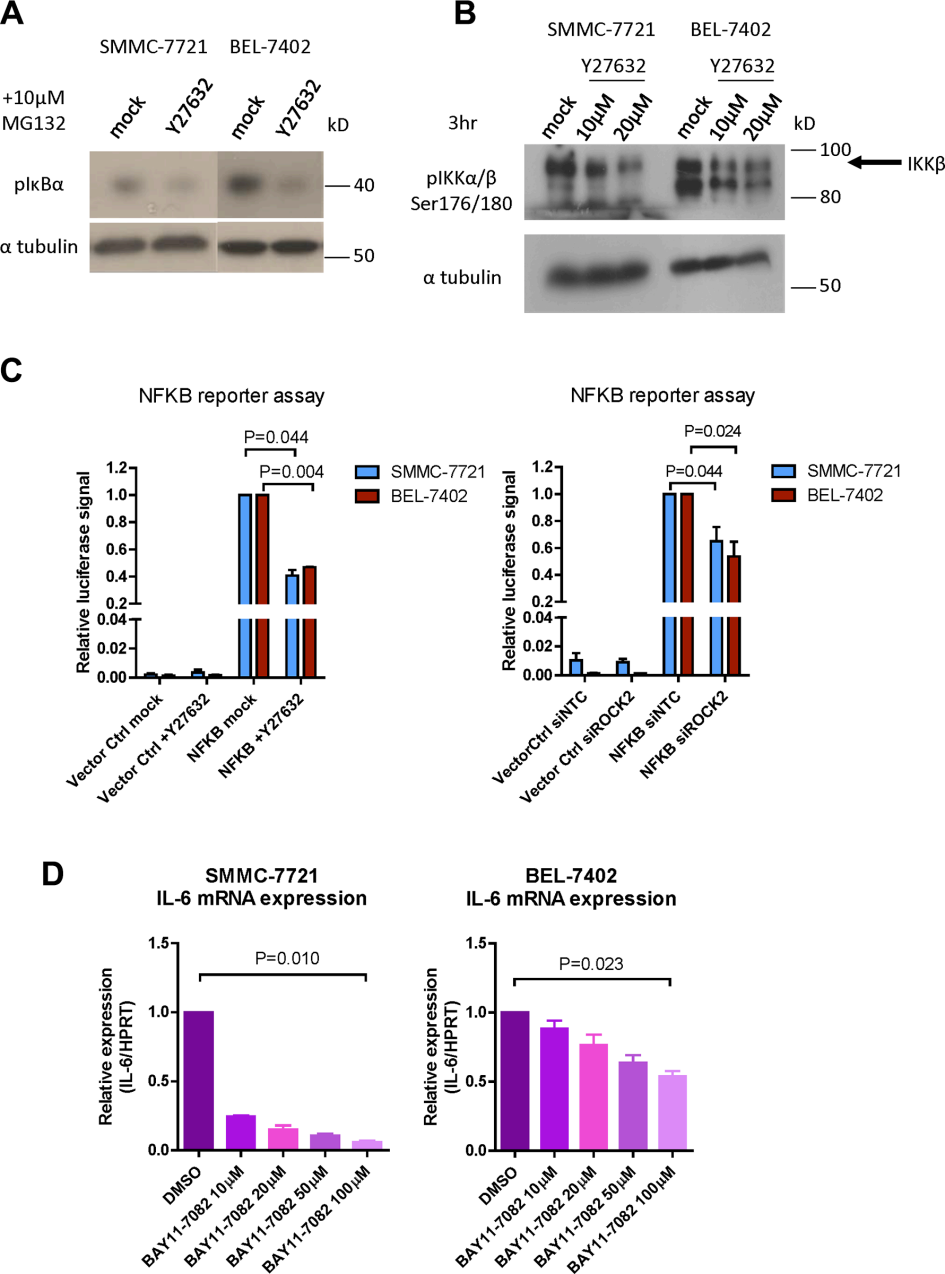
Rho/ROCK promotes IL-6 transcription via activation of IKKβ/NF-κB (**A**) Proteasome inhibitor MG132 was added to inhibit the degradation of p-IκBα. p-IκBα was reduced by 24-hour treatment of 10 μM Y27632 in BEL-7402 and SMMC-7721 cells. (**B**) BEL-7402 and SMMC-7721 cells were treated with indicated concentrations of Y27632 for 3 hours. Phosphorylation of IKKβ was suppressed by Y27632 treatment. (**C**) Inhibition of ROCK by Y27632 and ROCK2 knockdown both repressed NF-κB-driven transcription of firefly luciferase. SV40-driven expression of Renilla luciferase was used as normalization control. (**D**) BEL-7402 and SMMC-7721 cells were treated with the indicated concentration of NF-κB inhibitor, BAY11-7082, for 4 hours. mRNA expression level of IL-6 was decreased by BAY11-7082 in a dose-dependent manner. In all panels, 3 experiments are represented. *P*-value was calculated using Student's *t* test.

Although we demonstrated that Rho/ROCK signaling regulated the chemoresistance in HCC through IL-6/JAK2/STAT3, the direct phosphorylation target of ROCK2 remained unrevealed. Recent study has reported that IKKβ is a direct phosphorylation target of ROCK [21]. Since IL-6 is a well-documented target of NF-κB [22], this suggests that Rho/ROCK may induce IL-6/JAK2/STAT3 pathway through direct phosphorylation of IKKβ and activation of NF-κB. To elucidate this possibility, we assessed the p-IκBα and p-IKKβ levels with Western blot analysis. We observed significant reduction of p-IκBα and p-IKKβ after Y27632 treatment in both SMMC-7721 and BEL-7402 cells (Figure 6A and 6B). We also compared transcriptional activation activity of NF-κB between ROCK-inhibited cells and their corresponding controls by firefly luciferase reporter assay. Both knockdown of ROCK2 by siRNA and treatment with Y27632 suppressed the firefly luciferase signal indicating repressed activity of NF-κB (Figure 6C). To further confirm our result, we also examined the activity of NF-κB in our ROCK2-overexpressing cells using firefly luciferase reporter assay. Both ROCK2-overexpressing clones displayed enhanced firefly luciferase signal as compared with to their corresponding vector controls (Supplementary Figure 7). To demonstrate that IL-6 was also a direct downstream target of NF-κB in HCC cells, we treated SMMC-7721 and BEL-7402 with NF-κB inhibitor BAY 11-7082 for a short period of time (4 hours). The result showed that inhibition of NF-κB for 4 hours already significantly reduced the mRNA level of IL-6 and in a dose-dependent manner (Figure 6D). On the other hand, addition of BAY 11-7082 for 4 hours had no observable effects on the mRNA expression level of either IL-6R or gp130 (Supplementary Figure 8A). Treatment of Y27632 decreased pIKKα/β at around 6 hours as compared with the untreated cells. On the other hand, the pJAK2 level was lower in the Y27632-treated cells after 12 hours and pSTAT3 was suppressed in Y27632-treated cells after 24 hours (Supplementary Figure 9). This indicates that the effect of NF-κB on IL-6 signaling is mainly due to the change in the expression of the ligand without affecting the expression of the receptor complex. These findings support that Rho/ROCK signaling upregulates the activity of IL-6/JAK2/STAT3 pathway via phosphorylation of IKKβ and activation of NF-κB.

### ROCK2 expression correlates with NF-κB and IL-6 activations in human HCCs

**Figure 7:**
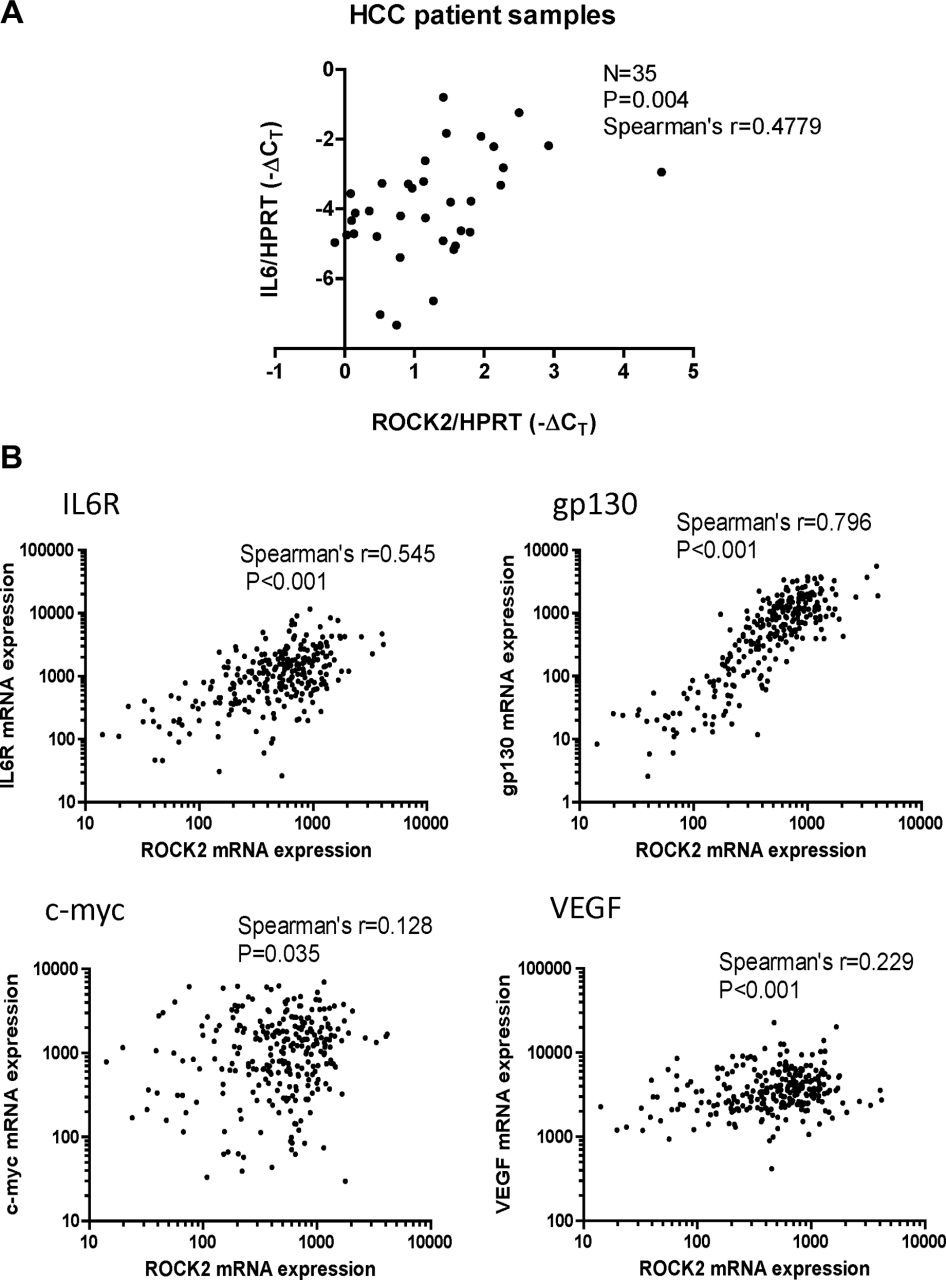
Clinical correlation of ROCK2 and activation of NF-κB and IL-6 signaling in HCC (**A**) The mRNA levels of ROCK2 and IL-6 in clinical HCC samples (*n* = 35) were measured by qPCR. HPRT was used as the endogenous control. ROCK2 expression was found to positively correlate with IL-6 expression with Spearman's *r* = 0.4779 (**B**) The mRNA expression levels of ROCK2 positively correlated with those of IL6R, gp130, c-myc and VEGF by Spearman's rank correlation in HCC patients' data from TCGA.

We then speculated that the relationship between ROCK signaling and activation of IL-6 and NF-κB was also of clinical significance. To test this hypothesis, we examined the mRNA levels of both ROCK2 and IL-6 in a cohort of 35 human HCC tissue samples by qPCR. We demonstrated that the IL-6 expression positively and significantly correlated with the ROCK2 expression in our sample cohort (Spearman's *r* = 0.4779, *P* = 0.004) (Figure 7A). Furthermore, we also utilized the data of HCC patients from the TCGA database. According to the TCGA data, ROCK2 expression positively and significantly correlated with those of IL6R, gp130, c-myc and VEGF in HCC patients (Figure 7B). This suggests that higher ROCK2 expression levels correlate with activation of IL-6 and NF-κB signaling in HCC patients.

## DISCUSSION

**Figure 8:**
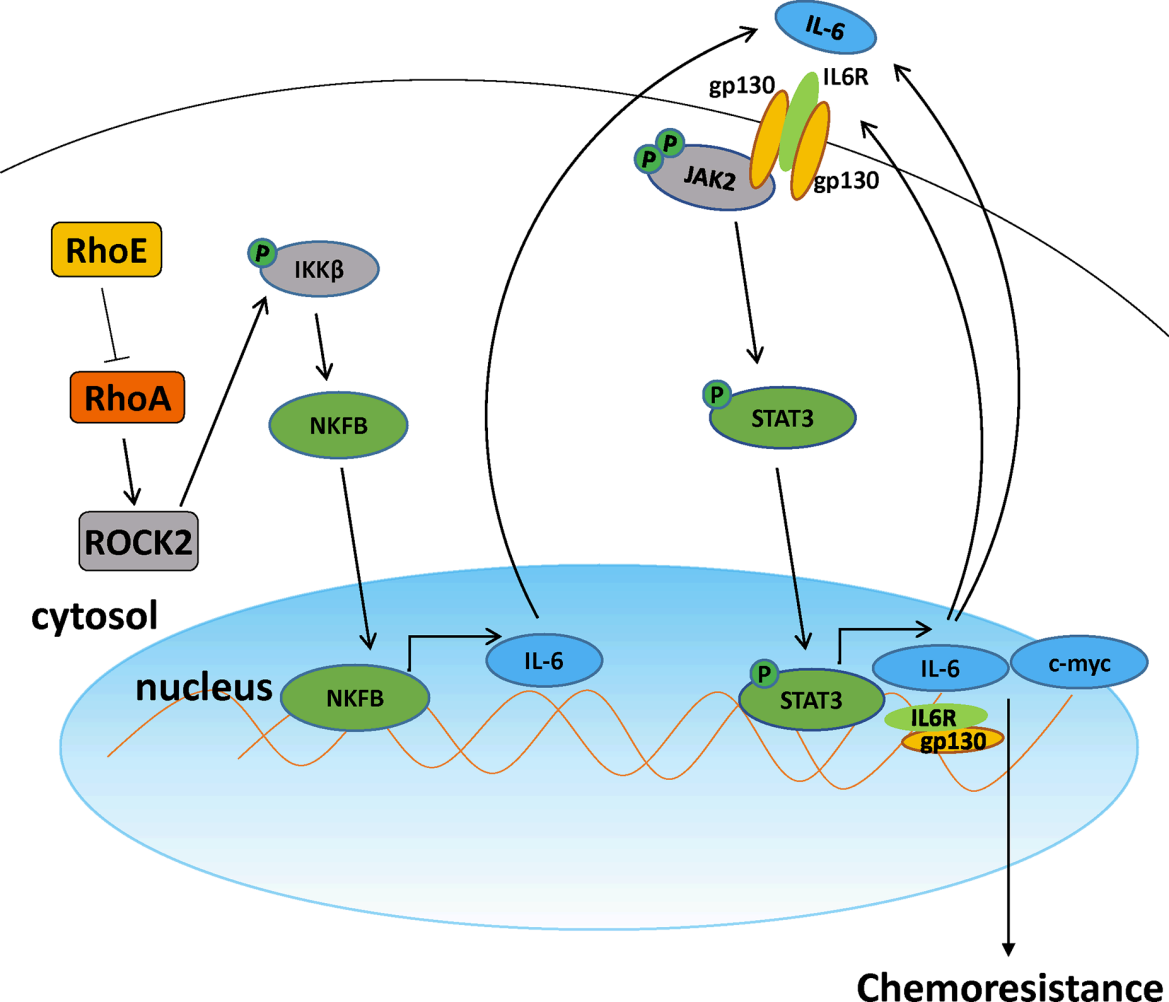
A schematic model of Rho/ROCK promoting chemoresistance in HCC

Our findings in this study have revealed that RhoE/ROCK2 signaling is one of the important regulators contributing to the high chemoresistance of HCC. In the model illustrated in Figure 8, we propose that both downregulation of RhoE and upregulation of ROCK2 lead to the augmentation of the ROCK2 activity. As a consequence, ROCK2 promotes the phosphorylation of IKKβ and activation of NF-κB, which then turns on the transcription of IL-6. IL-6 acts through an autocrine cycle, resulting in hyper-activation of STAT3. This in turn leads to the transcriptional activation of STAT3-specific target genes and promotion of cell survival. Our *in vivo* tumor growth study strongly suggests that inhibitors targeting ROCK2 have great potential to serve as a chemo-sensitizing therapy in HCC. In particular, the dramatic effect of combined Y27632 and cisplatin in inhibiting tumor growth over that of cisplatin alone in nude mice model indicates that the activation of ROCK2 may be crucial for maintaining the chemoresistance of HCC cells. Importantly, there was a positive association between the expression levels of ROCK2 and activation of IL-6 and NF-κB in HCC samples from patients. This further supports the clinical relevance of Rho/ROCK signaling in regulating chemoresistance, as IL-6 has been well documented to be a pro-survival cytokine in cancers [23].

Since ROCK has two isoforms, it is important to identify which one is specific and required for promoting chemodrug-resistance, in order to develop more specific inhibitors to improve targeting efficacy and reduce potential cytotoxicity. To our knowledge, although several studies have reported that inhibition of both ROCK1 and ROCK2 by pan-ROCK inhibitors could induce apoptosis in cancer cells [7, 15], the isoform-specific effect of ROCK1 and ROCK2 has not yet been explored. This is partially due to the reason that the two kinases share high homology in amino acid sequence (65%) and even higher homology in their kinase domains (95%). Despite the high similarity between the two isoforms, there is evidence suggesting they are not functionally redundant [13, 24]. By knocking down each isoform individually, we clearly demonstrated that only ROCK2 was responsible for promoting chemoresistance in HCC. We believed that two isoforms contribute differently in regulating apoptosis in HCC cells. It was demonstrated that inhibition of ROCK1 activation only abolish the fragmentation of apoptotic cells but does not halt the apoptotic process itself [25]. Based on our data, it is likely that in HCC only ROCK2 can protect the cells from entering apoptosis while ROCK1 only participates in late apoptotic events and has no effect on the initiation of apoptosis. It will be of interest to test whether ROCK2-specific inhibitors such as KD025 [26] serve as a better anti-metastatic and chemo-sensitizing therapy over the pan-ROCK inhibitors. Our team had previously shown that MHCC-97L cells expressed a lower level of miR-139, which is a ROCK2 expression inhibitor, than BEL-7402 and SMMC-7721 cells. Therefore it is not surprising to see that the chemo-sensitization effect of Y27632 was weaker in MHCC-97L cells than in BEL-7402 and SMMC-7721 cells. Hence a higher dose of Y27632 may be required in patients with higher endogenous ROCK2 activity.

Active RhoA has been reported to activate STAT3 by phosphorylation at both Y705 and S727 in HEK cells [11]. Nevertheless, the exact molecular mechanism of how Rho GTPase activates STAT3 signaling is likely to be cell-type dependent and remains unknown in HCC. Sanz-Moreno et al. demonstrated that in melanoma cells and the cancer-associated fibroblasts (CAFs), addition of pan-ROCK inhibitor H-1152 could suppress STAT3 phosphorylation through repression of JAK [27]. In another report, Huang et al. also identified JAK2 to be a direct target of ROCK1 in hypothalamic cells [28]. In these two reports, JAK phosphorylation was immediately abolished following inhibition of ROCK within 30 minutes, indicating that Rho/ROCK controls STAT3 signaling via direct manipulation of p-JAK levels. In contrast, in our study, the effect of Y27632 on suppressing the phosphorylation of STAT3 in HCC cells was only observed after at least 24 hours of incubation. Therefore JAK is unlikely to be the direct downstream target of ROCK in HCC and the effect of STAT3 activation following ROCK activation may be mediated by the increase in expression of autocrine IL-6, as in the case of Rac1-regulated STAT3 activation [29].

NF-κB is one of the most well-studied regulators of IL-6 expression [30]. Very recently, ROCK has been reported to directly phosphorylate IKKβ [21]. It is reasonable to hypothesize that ROCK mediates IL-6/JAK2/STAT3 activation through direct regulation of IKK phosphorylation. In support of this, our result showed that transient inhibition of ROCK for 3 hours could already reduce phosphorylation of IKKβ in HCC cells. This suggests that the NF-κB pathway is likely the direct effector of Rho/ROCK signaling. NF-κB activation leads to accumulation of IL-6 which in turn activates JAK2 and STAT3. Enhanced expression of IL-6R and gp130 should be a subsequent event of STAT3 activation since they are putative transcription targets of STAT3 [31, 32]. The activated STAT3 may then induce more transcription of IL-6, which forms a positive feedback loop and confer hyper-chemoresistance to HCC cells [33].

Currently, no ROCK inhibitors are approved for use to treat human cancers. Nevertheless, there is growing evidence that ROCK inhibition suppresses both cancer progression and metastasis in murine models [20, 34]. In this study, we have further discovered that ROCK2 activation is necessary for maintaining the highly chemoresistant nature of HCC. It is conceivable that ROCK2 is an attractive therapeutic target in HCC, since ROCK2 inhibitors may serve as both anti-metastasis and chemo-sensitizing agents in HCC patients.

## MATERIALS AND METHODS

### Cell lines, reagents and plasmids

Human HCC cell lines, SMMC-7721 and BEL-7402 and immortalized normal liver cell line LO2 were obtained from Shanghai Institute of Cell Biology, Chinese Academy of Sciences. MHCC-97L was a gift from Fudan University (Dr. ZY Tang) of Shanghai. BEL-7402 and MHCC-97L cells are hepatitis B surface antigen (HBsAg) positive. SMMC-7721, BEL-7402, MHCC-97L and LO2 were maintained in Dulbecco's modified Eagle's medium with high glucose (Gibco-BRL, Carlsbad) supplemented with 10% fetal bovine serum. Y27632, MG132, AG490 and BAY11-7082 were purchased from Selleckchem (Houston). Cisplatin and doxorubicin were purchased from Pharmachemie BV (Haarlem). The plasmids for STAT3 and NFκB luciferase reporter assays were constructed by cloning STAT3 and NFκB binding elements into pGL3(Promega, Wisconsin) using NheI and BgIII.

### Real-time quantitative PCR (q-PCR)

RNA was extracted with TRIzol reagent (Invitrogen, Carlsbad). cDNA was synthesized with 1 μg of total RNA using the Gold RNA PCR core kit (GeneAmp, Carlsbad) and random primers. Quantitative PCR (q-PCR) was performed in triplicate, using the SYBR^®^ Green Real-Time PCR Master Mixes and the Applied Biosystems 7900HT Fast Real-Time PCR system (Applied Biosystems, Carlsbad) according to manufacturer's instructions.

### Drug response assay

3 × 10^6^ cells were seeded in 35-mm culture plates 24 hours prior to experiment. Cells were replenished with 2 mL fresh culture medium containing 20 μg/mL cisplatin or 2 μg/mL doxorubicin and incubated at 37°C for 48 hours. After 48 hours both dead flowing cells and live cells were collected, resuspended in 1 mL PBS and were stained with Trypan Blue. Live cells and dead cells were counted by hematocytometer.

### Annexin V apoptosis assay

3 × 10^6^ cells were seeded in 35-mm culture plates 24 hours prior to the experiment. Cells were replenished with 2 mL fresh culture medium containing 20 μg/mL cisplatin for 24 hours. After 24 hours, both dead flowing cells and live cells were trypsinized and collected. Annexin V Apoptosis Detection Kit I (BD Biosciences, San Jose) was used according to manufacturer's protocol. The stained cells were analyzed by BD FACSCantoII Analyzer (BD Biosciences).

### Xenograft assay

6-week-old BALB/c-nu/nu athymic male nude mice were used. All animals were provided by the Laboratory Animal Unit of the University of Hong Kong. 2 × 10^6^ cells resuspended in 100 μL PBS were injected subcutaneously into both sides of the mice's back. For subcutaneous assay, tumor was allowed to grow until it reached 100 mm^3^. For orthotopic assay, tumor was allowed to grow for 1 week. After that, cisplatin and/or Y27632 were administered every 3 days to the mice via intraperitoneal injection. The tumor dimensions were also measured every 3 days. The volume of the tumor was estimated with the following formula: Tumor volume = shortest dimension × longest dimension^2^

### Luciferase assay

5 × 10^4^ cells were seeded in 24-well plate overnight. Renilla luciferase was used as the normalization control. Cells were transfected with 1 μg of total DNA of 1 (Renilla luciferase plasmid):100 (firefly luciferase reporter plasmid) using Lipofectamine 2000 (Invitrogen). Each sample was repeated in triplicate. 48 hours after transfection, luciferase assay was performed using the Dual-luciferase Reporter Assay System (Promega) according to the manufacturer's protocol. The luminescence was measured by Infinite 200 (Tecan, Männedorf).

### TCGA datasets

We collected transcriptomic data of HCC patients from The Cancer Genome Atlas (TCGA). Gene expression data (RNA-seq) were downloaded from cBioPortal [35]. Co-expression analysis was performed using Prism5.

### Statistics

Results are reported as mean ± SEM. Significance was tested by 2-tailed Student's *t* test. Mann–Whitney *U* test was used to compare luciferase signal in lung in xenograft mdoel. Cell proliferation rate and tumor growth rate were tested by non-linear regression test. Gene correlation was determined by Pearson correlation. A *P* value of less than 0.05 was considered significant.

### Study approval

Use of human HCC samples was approved by the Institutional Review Board of the University of Hong Kong/Hospital Authority Hong Kong West Cluster. Written informed consent for participation in the study was obtained from all participants. The procedures for collecting and using tissues from the patients were in accordance with the ethical standards established in the Declaration of Helsinki. All animal experiments were approved by the Committee on the Use of Live Animals in Teaching and Research, the University of Hong Kong and performed according to the Animals (Control of Experiments) Ordinance (Hong Kong) and the Institute's guidance on animal experimentation.

Additional methods can be found in Supplementary Methods.

## SUPPLEMENTARY MATERIALS



## Abbreviations

HBV: hepatitis B virus
HCC: hepatocellular carcinoma
Rho: Small Rho GTPase
ROCK: Rho-associated protein kinase
IL-6: interleukin-6.
