# Supplementary material for: RhoE/ROCK2 regulates chemoresistance through NF-κB/IL-6/ STAT3 signaling in hepatocellular carcinoma
Source: Oncotarget. 2016 May 18;7(27):41445–59. doi: 10.18632/oncotarget.9441 (PMC5173071; doi:10.18632/oncotarget.9441)
Supplement: Supplementary file 1 [file oncotarget-07-41445-s001.pdf]

RhoE/ROCK2 regulates chemoresistance through NF-κB/IL-6/STAT3 signaling in hepatocellular carcinoma

Supplementary Materials

MATERIALS AND METHODS

Antibodies

The following commercially available antibodies were used: Cell signaling: anti-RhoE(#3664), anti-pSTAT3(#9145), anti-STAT3(#4904), anti-pJAK2(#3776), anti-JAK2(3230), anti-Survivin(#2802), anti-pIKKα/β(#2697) and anti-pIκBα(#9246). Santa Cruz Biotechnology: anti-ROCK1(sc-5560), anti-ROCK2(sc-5561), anti-c-myc(sc-47694), anti-α tubulin(sc-53646) and anti-β actin(sc-47778). Cytoskeleton, Inc.: anti-RhoA(ARH03-A)

Establishment of stable knockdown cells

Small-hairpin RNAs were ordered from Sigma with targeting sequences stated below:

shRhoE-16: GATCCTAATCAGAACGTGAAA

shROCK1-59: GAGGTAAATGAACACAAAGTA

shROCK1-60: CCCGATTTAAGTAGTGACATT

shROCK2-78: GCCTTGCATATTGGTCTGGAT

Stable knockdown cells were established as previously described (1).

Immunohistochemistry (IHC)

IHC of formalin-fixed, paraffin-embedded samples was performed as described previously (2).

ELISA

Human IL-6 ELISA Kit (Shanghai ExCell Biology, Inc) was used according to manufacturer's protocol. 3×10^5 cells were seeded in 35-mm plates overnight. Cells were treated with either PBS or 10 µM Y27632 for 24 hours. Culture medium was collected and centrifuged at 12,000 × g for 5 minutes to remove cell debris. Result was measured at OD450 by Infinite 200 (Tecan).

Oligonucleotides

siRhoE, siROCK1, siROCK2 and siRhoA were purchased from Dharmacon as SMARTpool siRNA. The following primers were used for mRNA analysis by qPCR:

Target	Forward sequence	Reverse sequence
HPRT	TGCTCGAGA TGTGATGAAGG	TCCCCTGTTGACT GGTCATT
IL-6	TCCAGTTGC CTTCTTGGGAC	GTACTCCAGAAGAC CAGAGG
gp130	AGCGGCCA GAAGATCTACAA	CCCTCAGTACCTGG ACCAAA
IL6R	CTCCTGCCAG TTAGCAGTCC	TCTTGCCAGGTGAC ACTGAG

Establishment of stable TALE-TF expressing cells

All the plasmids and reagents for assembling the TAL effectors were from the Zhang Lab TALE Toolbox (cat#1000000019, Addgene). The effectors were assembled as described (3). The targeting sequence was designed using TAL effector Resources Center (www.taleffectors.com). The targeting sequence of ROCK2 is TAGCGGCGGCGCGCGGCC. For establishment of stable TALE-TF expressing cells, 2×10^5 cells were first seeded in 6-well plates overnight. 2 µg of TALE-TF plasmid was transfected with 5 µL Lipofectamine 2000. After 48 hours of transfection, positive TALE-TF expressing cells were selected by adding 400 µg/mL hygromycin B. The cells were selected for 5 days and monoclones were isolated.

HCC patient samples analysis

Thirty-five Chinese patients who had surgical resection at Queen Mary Hospital of Hong Kong from 1991 to 2000 were randomly selected for study (27 men and 8 women; age ranging from 35 to 74 years). Twenty-four (68.6%) of the 35 HCC patients were serum hepatitis B surface antigen (HBsAg) positive, whereas 2 (5.71%) patients were serum anti-hepatitis C virus (anti-HCV) positive. Their resected specimens were collected at the time of surgical resection, snapped frozen in liquid nitrogen, and kept at −80°C. The mRNA levels of IL-6 and ROCK2 were measured by qPCR using Taqman probes (Applied Biosystems). Use of human samples was approved by the institutional review board of the University of Hong Kong/Hospital Authority Hong Kong West Cluster (UW 09-185).

Statistics

Results are reported as mean ± SEM. Significance was tested by 2-tailed Student's *t* test. Tumor growth rate was tested by non-linear regression test. Clinical correlation was determined by Spearman's rank correlation. A *P*-value of less than 0.05 was considered significant.

REFERENCES

1. Ma W, Wong CCL, Tung EKK, Wong CM, Ng IOL. RhoE is frequently down-regulated in hepatocellular carcinoma (HCC) and suppresses HCC invasion through antagonizing the Rho/Rho-Kinase/Myosin phosphatase target pathway. Hepatology. 2013; 57:152–161.
2. Lee TK, Castilho A, Cheung VC, Tang KH, Ma S, Ng IO. CD24(+) liver tumor-initiating cells drive self-renewal and tumor initiation through STAT3-mediated NANOG regulation. Cell. Stem Cell 2011; 9:50–63.
3. Sanjana NE, Cong L, Zhou Y, Cunniff MM, Feng G, Zhang F. A transcription activator-like effector toolbox for genome engineering. Nat Protoc. 2012; 7:171–192.

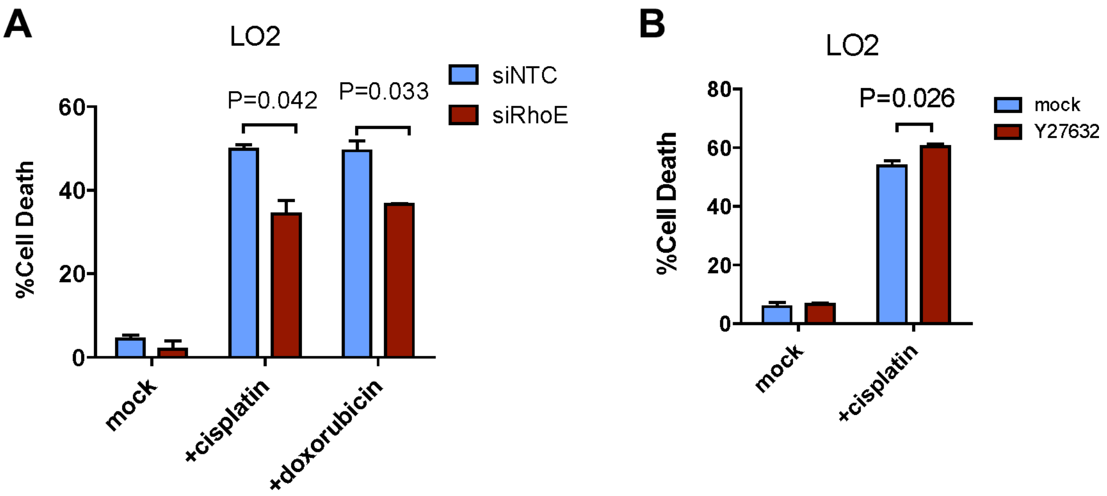

Supplementary Figure S1: RhoE/ROCK signaling regulated chemoresistance in immortalized normal liver cells. (**A**) Knockdown of RhoE by siRNA in LO2 cells protected them from cispatin and doxorubicin-induced cell death. (**B**) Treatment of ROCK inhibitor Y27632 slightly sensitized LO2 cells to cisplatin-induced cell death.

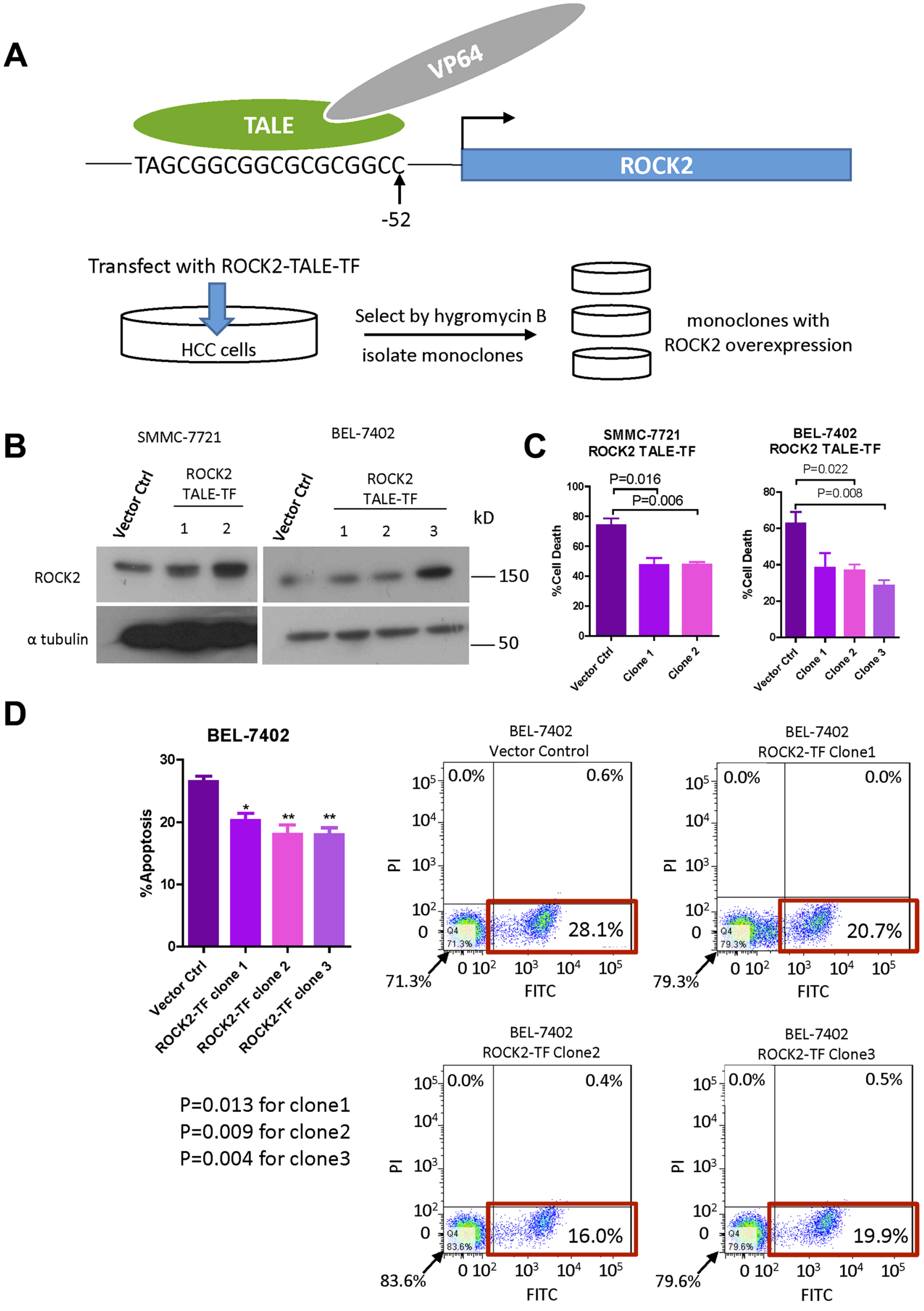

Supplementary Figure S2: Overexpression of ROCK2 conferred chemoresistance in HCC cells. (**A**) Schematic diagram showing the principle of TALE-mediated ROCK2 overexpression system and the process of how ROCK2-overexpressing monoclones were established. (**B**) Western blot analysis confirmed the successful overexpression of ROCK2 in BEL-7402 and SMMC-7721 cells. (**C**) Quantification of the percentages of cell death induced with 48-hour treatment of cisplatin in ROCK2-overexpressing clones. (**D**) ROCK2 overexpression suppressed cisplatin-induced apoptosis in BEL-7402 and SMMC-7721 cells. In all panels, 3 experiments are represented. *P*-value was calculated using Student's *t* test.

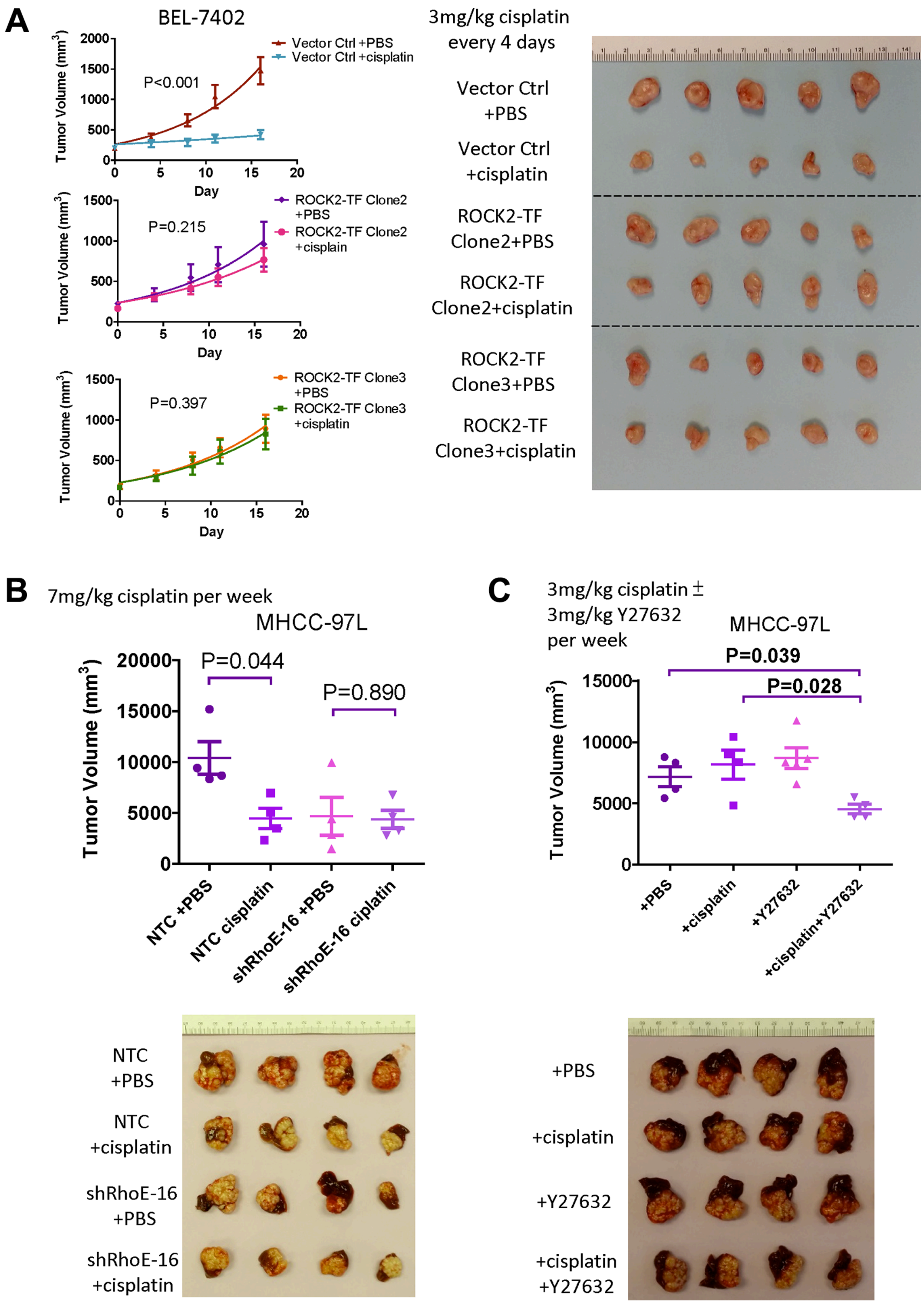

Supplementary Figure S3: ROCK2 promotes chemoresistance of HCC *in vivo*. (**A**) Subcutaneous xenograft tumor model was performed using ROCK2-overexpressing BEL-7402 cells. Cisplatin suppressed the tumor growth of vector control cells only, while ROCK2-overexpressing clones showed no observable response to cisplatin treatment. (**B** and **C**) Orthotopic xenograft model was used. HCC cells were injected directly to the left lobe of liver in nude mice. Tumors were allowed to grow for a week and then the mice were given the indicated treatment. (B) RhoE knockdown MHCC-97L cells and NTC were used. Only growth of NTC tumors showed significant inhibition by cisplatin. (C) Parental MHCC-97L cells were used. Mice were given i) PBS, ii) 3 mg/kg cisplatin, iii) 3 mg/kg Y27632, and iv) 3 mg/kg cisplatin+3 mg/kg Y27632 weekly. Combined treatment of cisplatin and Y27632 showed higher efficacy in suppressing tumor growth than cisplatin alone. The representative result from 3 experiments is shown. *P*-value was calculated using non-linear regression.

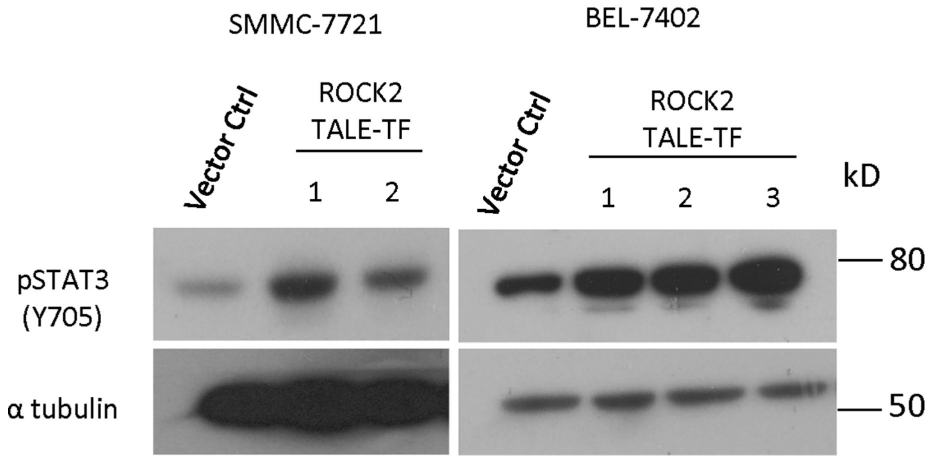

Supplementary Figure S4: ROCK activity correlated with STAT3 activation *in vitro*. ROCK2 overexpression upregulated the phosphorylation of STAT3 at Y705 in both BEL-7402 and SMMC-7721 cells.

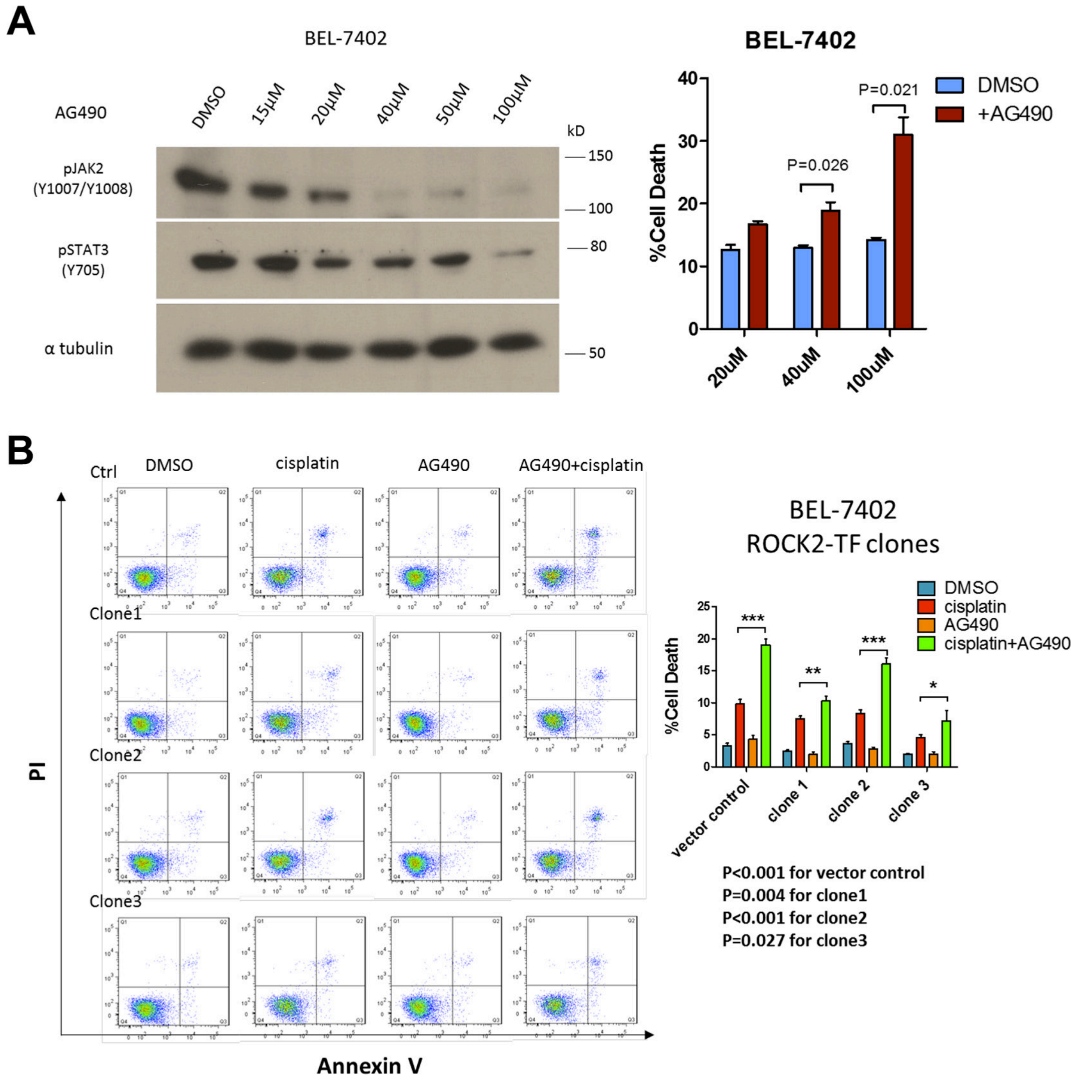

Supplementary Figure S5: Inhibition of JAK2 sensitized HCC cells to cisplatin. (**A**) Treatment with JAK2 inhibitor, AG490, for 24 hours repressed the phosphorylation JAK2 and STAT3 in a dose-dependent manner. In addition, BEL-7402 cells were sensitized to cisplatin treatment by co-treatment with AG490. (**B**) Treatment with AG490 rescued the enhanced chemoresistance of ROCK2 overexpressing BEL-7402 cells. The percentage of cell death was represented by the percentage of Annexin V^{+ve} cells. Three experiments are represented. *P*-value was calculated using Student's *t* test.

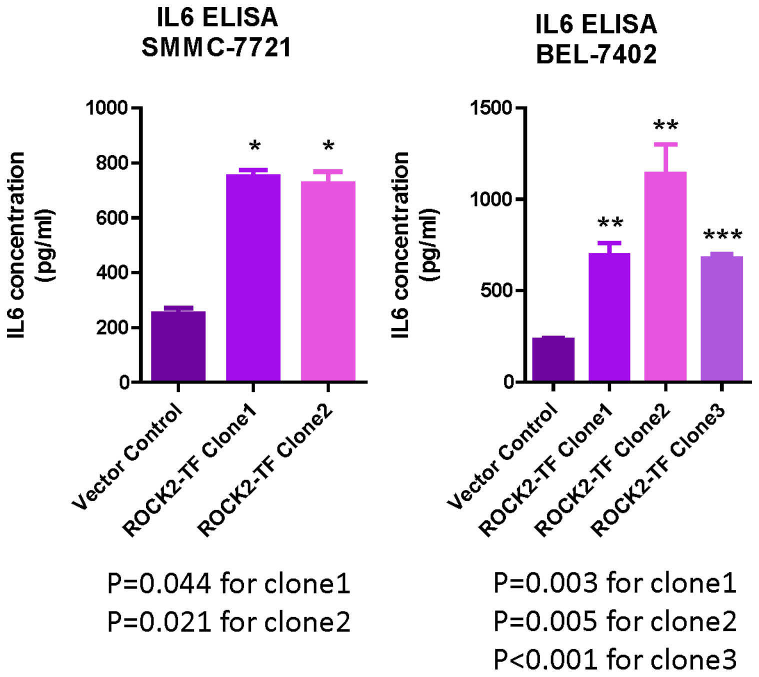

Supplementary Figure S6: Overexpression of ROCK2 enhanced IL-6 expression in HCC cells. qPCR was used to measure the IL-6 mRNA level in ROCK2-overexpressing BEL-7402 and SMMC-7721 cells. IL-6 mRNA level was markedly elevated in ROCK2-overexpressing clones as compared to the corresponding vector controls. In all panels, 3 experiments are represented. *P*-value was calculated using Student's *t* test. **P* < 0.05.

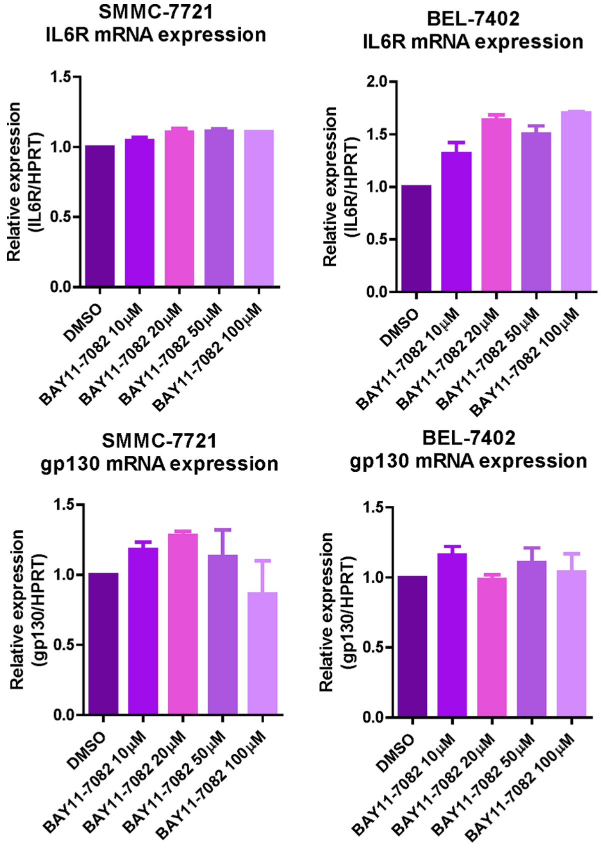

Supplementary Figure S7: Overexpression of ROCK2 activated NF-κB transcription activity. Luciferase reporter assay was used to assess the NF-κB transcription activity in ROCK2-overexpressing BEL-7402 and SMMC-7721 cells. SV40-driven expression of Renilla luciferase was used as normalization control. Overexpression of ROCK2 significantly promoted NF-κB-driven firefly luciferase transcription. In all panels, 3 experiments are represented. *P*-value was calculated using Student's *t* test. **P* < 0.05, ***P* < 0.01, ****P* < 0.001.

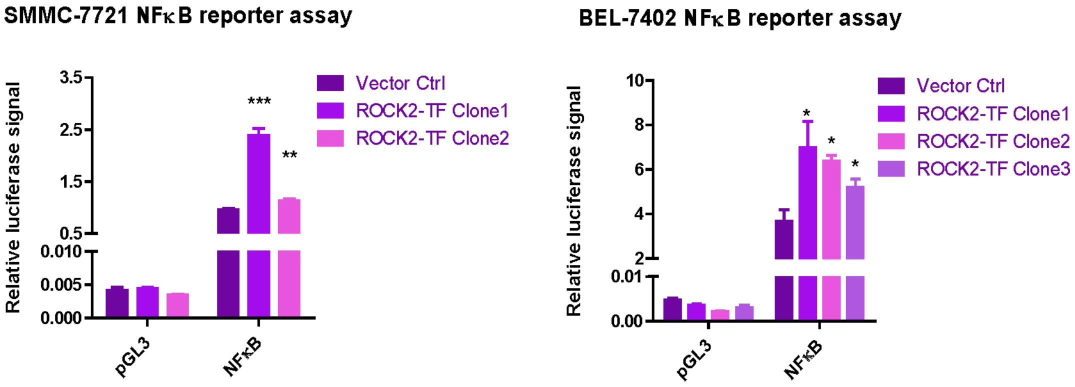

P=0.001 for clone1
P=0.009 for clone2

P=0.026 for clone1
P=0.021 for clone2
P=0.022 for clone3

Supplementary Figure S8: Inhibition of NFκB did not affect the expression of IL-6 receptor. Four hours of treatment with BAY11-7082 had no effect on the mRNA expression levels of IL-6R and gp130 in BEL-7402 and SMMC-7721 cells. In all panels, 3 experiments are represented.

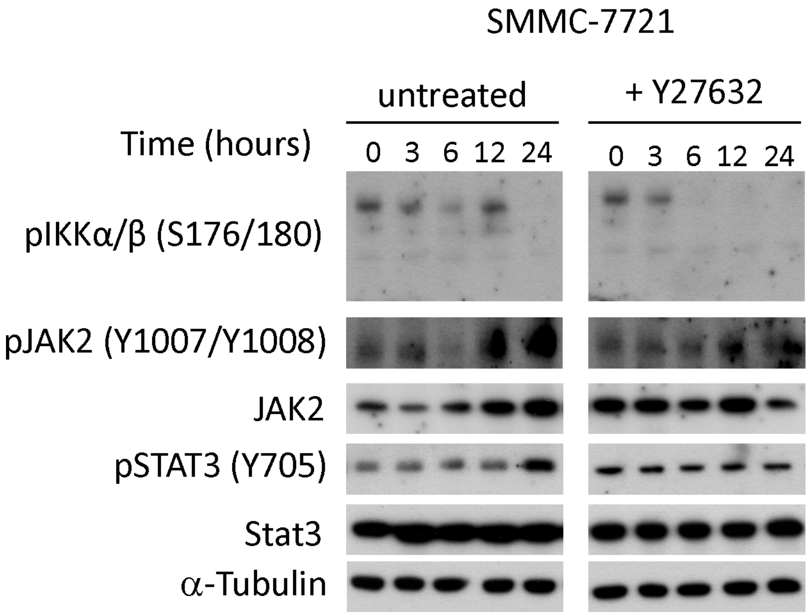

Supplementary Figure S9: Inhibition of ROCK suppressed activation of IKKα/β, JAK2 and STAT3 in a sequential manner. Treatment of Y27632 decreased pIKKα/β at around 6 hours as compared with the untreated cells. The pJAK2 level was lower in the Y27632-treated cells after 12 hours and pSTAT3 was suppressed in Y27632-treated cells after 24 hours.
